# Screening for Obesity in Reproductive-Aged Women

**Published:** 2011-10-15

**Authors:** Chloe Zera, Susan McGirr, Emily Oken

**Affiliations:** Division of Maternal-Fetal Medicine, Department of Obstetrics, Gynecology and Reproductive Biology, Brigham and Women’s Hospital; Harvard Medical School, Boston, Massachusetts; Obesity Prevention Program, Department of Population Medicine, Harvard Medical School and Harvard Pilgrim Health Care, Boston, Massachusetts

## Abstract

Although obesity screening and treatment are recommended by the US Preventive Services Task Force, 1 in 5 women are obese when they conceive. Women are at risk for complications of untreated obesity particularly during the reproductive years and may benefit from targeted screening. Risks of obesity and potential benefits of intervention in this population are well characterized. Rates of adverse pregnancy outcomes including gestational diabetes, preeclampsia, cesarean delivery, and stillbirth increase as maternal body mass index increases. Offspring risks include higher rates of congenital anomalies, abnormal intrauterine growth, and childhood obesity. Observational data suggest that weight loss may reduce risks of obesity-related pregnancy complications. Although obesity screening has not been studied in women of reproductive age, the effect of obesity and the potential for significant maternal and fetal benefits make screening of women during the childbearing years an essential part of the effort to reduce the impact of the obesity epidemic.

## Introduction

In 2009, 26% of US adult women reported a body mass index (BMI) in the obese range (≥30 kg/m^2^) (1). With rates of both adult and adolescent obesity increasing, the prevalence of obesity among women of childbearing age (aged 15-44 y) can also be expected to increase. The US Preventive Services Task Force (2) and the National Heart, Lung, and Blood Institute (NHLBI) (3) recommend screening all adults for obesity in clinical settings, but neither specifically mentions screening for women during their reproductive years. Although the Centers for Disease Control and Prevention's Select Panel on Preconception Care released recommendations in 2006 to improve the preconception care of women, which included addressing prepregnancy obesity, obesity remains an undertreated chronic disease that affects at least 1 in 5 pregnancies (4). One factor contributing to the gap between recommendation and practice may be that for the 20% of women of childbearing age who are uninsured, preventive care services are often provided episodically in settings such as federally funded family planning programs rather than in traditional primary care settings.

The effect of untreated obesity among women of childbearing age includes adverse reproductive outcomes as well as adverse outcomes for these women's offspring. Although women who are obese may be at increased risk for unplanned pregnancy and contraceptive failure (5), infertility rates are higher among obese women than among normal-weight women (6). Once a woman is pregnant, both maternal and fetal risks are increased by high maternal BMI. Pregnancy-associated morbidity and mortality are higher in obese women than in normal-weight women (7). The offspring of obese women face an increased risk of obesity and other chronic metabolic diseases (8). Women of childbearing age are therefore a uniquely at-risk population who may benefit from targeted screening.

Our objective was to develop recommendations for screening in women of childbearing age by focusing on the efficacy, benefits, and potential harms of screening in this population. This article is part of a series of articles focused on screening women of reproductive age for chronic health conditions, particularly women seeking care at clinics that receive Title X Family Planning funding.

We systematically reviewed the literature on obesity screening in women of reproductive age. We searched the MEDLINE database for articles published from January 1, 1998, to July 1, 2010. We used the search terms "obesity" or "BMI" and "screen" or "assessment" limited to humans aged 19 to 64 years to identify potentially relevant articles. Using these parameters, we were unable to identify any studies that focused on the feasibility, acceptability, risks, benefits, or costs of obesity screening in women of childbearing age. We therefore chose to frame our discussion of screening by summarizing the current evidence surrounding the risks of obesity before and during pregnancy, reviewing potential risks and benefits of obesity treatment as part of preconception care, highlighting gaps in current screening practices, and proposing recommendations for screening opportunities in the population of women who may become pregnant.

## Risks Associated With Obesity in Women of Childbearing Age

Although unplanned pregnancy is of concern in a population at high risk for complications during pregnancy, obese women are less likely to use contraception than normal-weight women (9). Furthermore, the counseling of obese women regarding contraceptive options is more complex than for women of normal weight. Although contraceptive efficacy is generally high, there may be higher rates of failure of combined hormonal methods in obese women (10), and both intrauterine contraception placement and surgical sterilization are more technically challenging and more likely to result in complications (11) in women with a high BMI than in women of normal weight. Although the absolute risk of venous thromboembolism is small in obese women who use combined hormonal contraception, the risk is elevated relative to women who are not using combined contraception, and further study is needed to determine whether risks are higher in obese women than in normal-weight women (5).

The risks of adverse pregnancy outcomes increase as maternal BMI increases beyond the normal range (BMI ≥25 kg/m^2^). Achieving pregnancy can be more difficult for obese women because they are less likely to ovulate regularly, have decreased fecundity, and have increased risk of miscarriage (6). Gestational diabetes affects as many as 20% of pregnancies in women who are obese, a 4-fold increase compared with normal-weight women (12). Hypertension in pregnancy is more frequent in obese women, and they have a 2- to 3-fold increased odds of preeclampsia (12). The risk of preterm delivery in obese women is also elevated, likely in part because of their increased risk of preeclampsia (12).

High maternal BMI is associated with intrapartum complications. Induction of labor is more common in women with high BMI than in normal-weight women (12). Rates of both primary and repeat cesarean delivery increase with increasing maternal BMI, with trial of labor after cesarean less likely to be successful than in normal-weight women (12,13). The peripartum complications faced by obese women include a greater likelihood of infection, need for blood transfusion, and venous thromboembolism, particularly with cesarean delivery (14). Prepregnancy obesity is also associated with a 3-fold increase in maternal death and near-miss morbidity (7).

In addition to the maternal consequences of obesity, there are significant implications of excess maternal weight to offspring. The risk of fetal anomalies including cleft palate, neural tube defects, and congenital heart disease is increased (15), and detection of anomalies is made more difficult because of the technical challenges of ultrasound in obese women. The risk of stillbirth among obese women is approximately double that among normal-weight women (16). Obese women are more likely to give birth to infants who are large for gestational age or macrosomic (14); these infants are at higher risk than their normal birth weight counterparts for long-term cardiometabolic complications, including obesity (8). Even normal birth weight offspring of obese mothers are more likely to be obese than offspring of normal-weight mothers (8), further fueling the obesity epidemic.

## Preconception Interventions

The NHLBI has recommended treating all obese patients who are receptive to intervention, while acknowledging that a limitation of all therapies, including lifestyle intervention, medical therapy, and surgical therapy, is a low rate of long-term weight maintenance (3). Nonetheless, randomized controlled trials of multiple weight-loss strategies have demonstrated short-term efficacy. None of these trials has specifically focused on the treatment of obesity during a woman's reproductive life, particularly before conception. Observational data suggest that interpregnancy weight loss is associated with a decreased risk of preeclampsia, large-for-gestational-age birth weight, and cesarean delivery (17), compared with the previous pregnancy. Few studies have examined the effect of lifestyle interventions or medical therapy before conception on pregnancy outcomes. Several case series have attempted to examine the effect of bariatric surgery by comparing outcomes in women who have had pregnancies both pre- and postoperatively, or by choosing normal-weight controls. There may be a decrease in infertility and spontaneous abortion in women who have undergone bariatric surgery (5). Consistent findings include lower rates of gestational diabetes and hypertensive complications (18) after surgical intervention, without increases in fetal or maternal morbidity. One study of children of mothers who had undergone bariatric surgery found that, compared with children born before their mothers had bariatric surgery, fewer children born after their mothers had bariatric surgery showed abnormalities in anthropometry and hormonal markers of cardiometabolic risk (19). While these data suggest that aggressive treatment of obesity may be warranted, prospective study is needed to quantify the risks and benefits of both medical and surgical management before pregnancy.

## Obesity Screening

The rate of obesity screening in clinical practice is low. Obesity is underdiagnosed in primary care settings (20); consistently fewer than half of obese patients have a documented obesity diagnosis. Similarly, a minority of obese people report receiving weight-loss advice from their health care provider (21). Although some clinicians have argued that screening is unnecessary because patients have an adequate perception of body weight, misperception of weight status is in fact widespread, with 1 study reporting that only 14% of obese women recruited from urban health centers in Atlanta correctly identified their weight status (22).

Theoretical risks of obesity screening include stigmatization or psychological effects associated with the diagnosis of obesity; however, we were unable to identify literature substantiating these risks. In fact, data suggest that awareness of weight status is beneficial. The 2003-2008 National Health and Nutrition Examination Survey found that 98% of the women who correctly perceived themselves as overweight desired to weigh less and 72% were actively attempting to lose weight, compared with only 37% of women who did not perceive themselves as overweight (23). Moreover, overweight and obese women who had received a diagnosis of overweight or obesity from their health care provider were twice as likely to endorse weight control behaviors (ie, diet, exercise) as women who did not have a formal diagnosis (23). The potential benefits of obesity screening as a part of preconception counseling remain unexplored.

## Recommendations for Screening

Screening for obesity in women of reproductive age remains understudied as a population-level intervention. There are data, however, that support the basic tenets of screening for obesity in women during childbearing years. Obesity is a disease with measurable public health impact during the reproductive years, not just later in life. Screening for obesity by calculating BMI is a reproducible, simple, and inexpensive technique that reliably estimates adiposity without the time and expense of less practical methods such as measurement of total body water (8). Although there are theoretical risks, including misdiagnosis, stigma associated with diagnosis, and extra cost associated with treatment, these risks have not been demonstrated in current literature.

Finally, the potential benefits of screening include appropriate treatment with resultant weight loss, which may improve outcomes for women and their children. In particular, weight loss before pregnancy may have immediate benefits including reduced maternal morbidity and decreased risk of long-term cardiometabolic consequences for children, even if weight loss is not sustained over time. At a minimum, identification of obesity before conception allows for appropriate preconception counseling. Current recommendations from the American Congress of Obstetricians and Gynecologists include discussion of specific maternal and fetal risks of prepregnancy obesity, counseling regarding the benefits of weight reduction, and management to identify early or minimize possible complications of obesity (24).

We are unaware of prior reviews of obesity screening that have focused on women of reproductive age. Although we were not able to identify sufficient literature regarding obesity screening to perform a meta-analysis, evidence regarding the risks of untreated obesity in women of reproductive age is substantial. The limited data on pregnancy outcomes following the surgical treatment of obesity are promising and suggest there may be benefit to identifying and treating obesity in women who are planning pregnancy.

The American Congress of Obstetricians and Gynecologists is the only national body that has issued specific guidelines for screening women of reproductive age for obesity, with the recommendation that BMI be calculated for all women (24). Despite these recommendations, current screening rates are low (20). Recognizing that many young women access the health care system sporadically and that nearly 50% of pregnancies are unplanned, we believe that screening should be performed in settings beyond primary care clinics, including community health centers, college health service centers, and family planning clinics. We suggest that women of childbearing age presenting for care in these settings should be told their BMI after a measured weight and height are obtained.

The potential benefits of screening all women of childbearing age include identification of women at high risk and referral to appropriate treatment before pregnancy. The American Dietetic Association and the American Society for Nutrition have issued a joint statement supporting counseling all overweight and obese women of reproductive age on dietary modification and physical activity (25). Further data are needed to understand the effect that losing a small amount of weight has on pregnancy outcomes. A potential harm is stigma, although this risk has not been studied, and additional costs may be incurred with increased diagnosis of obesity. In resource-poor settings, follow-up care may be inadequate to ensure appropriate treatment of women identified in broad screening programs. Although we acknowledge these potential pitfalls of widespread screening, we argue that simply identifying obesity may itself be an intervention because women who accurately perceive that they are overweight may be more likely to change their diet or demonstrate healthy weight-related behaviors than women who misperceive their weight status (23).

## Conclusions

Although obesity screening has not been studied specifically in women of reproductive age, there are compelling reasons to prioritize screening in this population. Women of childbearing age who are obese are at increased risk of infertility, miscarriage, and other adverse pregnancy outcomes. Mounting evidence supports the intergenerational transmission of obesity as a result of an abnormal intrauterine environment. Although randomized controlled trials of intensive treatment of obesity before pregnancy have not yet been performed, observational data suggest the possibility that weight reduction may ameliorate these risks. Furthermore, clinicians who identify women as obese may be more likely to provide appropriate care and screening to identify and reduce risks for secondary complications of obesity. Screening of reproductive-aged women is an essential part of broadening efforts to reduce the effect of the obesity epidemic in this country.
